# The role of microRNAs in atopic dermatitis

**DOI:** 10.1016/j.ncrna.2024.05.012

**Published:** 2024-05-28

**Authors:** Mahsa Khosrojerdi, Farahzad Jabbari Azad, Yaser Yadegari, Hamid Ahanchian, Amir Azimian

**Affiliations:** a Department of Immunology and Allergy, Faculty of Medicine, Mashhad University of Medical Sciences, Mashhad, Iran; b Department of Pathobiology and Laboratory Sciences, Faculty of Medicine, North Khorasan University of Medical Sciences, Bojnurd, Iran

**Keywords:** Atopic dermatitis, microRNA, Autoimmune, Skin

## Abstract

Atopic dermatitis (AD), known as eczema, is a chronic inflammatory skin condition affecting millions worldwide. This abstract provides an overview of the clinical features and underlying pathogenesis of AD, highlighting the role of specific microRNAs (miRNAs) in its development and progression. AD presents with distinct clinical manifestations that evolve with age, starting in infancy with dry, itchy skin and red patches, which can lead to sleep disturbances. In childhood, the rash spreads to flexural areas, resulting in lichenification. In adulthood, lesions may localize to specific areas, including the hands and eyelids. Pruritus (itchiness) is a hallmark symptom, often leading to excoriations and increased vulnerability to skin infections. The pathogenesis of AD is multifaceted, involving genetic, immunological, and environmental factors. Skin barrier dysfunction, immune dysregulation, genetic predisposition, microbiome alterations, and environmental triggers contribute to its development. Recent research has uncovered the role of miRNAs, such as miR-10a-5p, miR-29b, miR-124, miR-143, miR-146a-5p, miR-151a, miR-155, and miR-223, in AD pathogenesis. These microRNAs play crucial roles in regulating various aspects of immune responses, keratinocyte dynamics, and inflammation. MicroRNA-10a-5p orchestrates keratinocyte proliferation and differentiation, while miR-29b regulates keratinocyte apoptosis and barrier integrity. MicroRNA-124 exhibits anti-inflammatory effects by targeting the NF-κB signaling pathway. MicroRNANA-143 counters allergic inflammation by modulating IL-13 signaling. MicroRNA-146a-5p regulates immune responses and correlates with IgE levels in AD. MicroRNA-151a shows diagnostic potential and modulates IL-12 receptor β2. MicroRNA-155 plays a central role in immune responses and Th17 cell differentiation, offering diagnostic and therapeutic potential. MicroRNA-223 is linked to prenatal smoke exposure and immune modulation in AD. Understanding these microRNAs' intricate roles in AD pathogenesis promises more effective treatments, personalized approaches, and enhanced diagnostic tools. Further research into these molecular orchestrators may transform the landscape of AD management, improving the quality of life for affected individuals.

## 1 Clinical features and pathogenesis of atopic dermatitis

Atopic dermatitis (AD), also known as eczema, is a chronic inflammatory skin condition that affects millions of people worldwide. It is characterized by distinctive clinical features and a complex underlying pathogenesis [1]. Atopic dermatitis presents with a range of clinical manifestations that often evolve with age [2]: (see Table 1)

**Table 1:** The features of microRNAs in Atopic Dermatitis.

MicroRNA	Expression in AD	Possible clinical application	mechanism	Target molecule	Target Cell
MiR-151a	↑	Diagnostic	Inhibition of IL12 signaling pathway	IL12RB2	T helper
MiR-155	↑	Diagnostic/Therapeutic	Elevation of Th-17 differentiation/elevation of T-cell activation	CTLA-4	T cells/Keratinocytes
MiR-10a-5p	↑	Therapeutic	Prevention of keratinocyte proliferation	HAS-3	Keratinocytes
MiR-29b	↑	Therapeutic	Increase of IFN-γ related apoptosis in keratinocytes	BCL2L2	Keratinocytes
MiR-124	↑	Therapeutic	Prevention of inflammatory responses	P65	Keratinocytes
MiR-143	↓	Therapeutic	Prevention of IL-13 related skin barrier damages	IL-13Rα1	Keratinocytes
MiR-146a-5p	↑	Therapeutic	Suppressing of NFKB related proinflammatory cytokines and chemokines/suppress IgE level	IRAK1, TRAF6, CARD10, CCL5	Keratinocytes
MiR-223	↑	Therapeutic	Correlated to T-reg number	–	T-reg

Infancy: AD frequently begins in infancy, with dry and itchy skin. Red, inflamed patches typically appear on the face, scalp, and extremities. Infants may exhibit intense itching, leading to sleep disturbances and fussiness [3].

Childhood: As children grow, the rash often spreads to flexural areas, such as the creases of elbows and knees. Repeated scratching can result in lichenification (thickened, leathery skin) in these regions. AD can also affect the hands and feet [4].

Adulthood: In adults, AD lesions may localize to specific areas, including the hands, feet, and eyelids. While the severity of AD may decrease with age in some individuals, it can persist throughout life [5].

Pruritus (Itchiness): Intense pruritus is a hallmark symptom of AD and can be debilitating. The urge to scratch can lead to excoriations, open sores, and a vicious cycle of worsening inflammation [6].

Susceptibility to Infections: AD patients are more susceptible to skin infections, particularly *Staphylococcus aureus* and *herpes simplex virus*. The disrupted skin barrier and immune dysregulation contribute to this vulnerability [7].

The of AD is intricate and involves a combination of genetic, immunological, and environmental factors [8]. AD is associated with abnormalities in the skin's barrier function. Reduced levels of essential proteins and lipids in the skin's outermost layer make it more permeable to allergens, irritants, and microbes [9]. Dysregulated immune responses play a central role in AD. Type 2 immune responses, characterized by the activation of T helper type 2 (Th2) cells and the production of cytokines such as IL-4, -5, -9, and -13, contribute to inflammation, pruritus, and tissue damage [10]. Genetic factors significantly influence an individual's susceptibility to AD. Variations in genes related to skin barrier function, immune regulation, and epidermal differentiation increase the risk of developing AD [11]. Changes in the skin's microbiome are observed in AD patients, often characterized by an overgrowth of *Staphylococcus aureus* [12]. Microbial imbalances can further exacerbate inflammation and skin barrier disruption [13]. Environmental factors such as allergens, pollutants, and climate can exacerbate AD symptoms. Allergen sensitization, particularly to common allergens like dust mites and pollen, perpetuates the inflammatory response [14]. Recent research has uncovered the role of microRNAs, small RNA molecules that regulate gene expression, in AD pathogenesis. microRNAs are involved in modulating key processes such as inflammation and epidermal differentiation [15].

Understanding both the clinical features and the underlying pathogenesis of AD is crucial for developing effective treatments. Current therapeutic approaches aim to restore the compromised skin barrier, manage inflammation, and alleviate pruritus. Ongoing research, including investigations into microRNA-based therapies, offers promise for improved AD management in the future. In this review, we evaluate the microRNAs with proven role in AD to date.

## 2 microRNAs: the molecular orchestrators

MicroRNAs (miRNAs) are a class of small RNA molecules that play a pivotal role in post-transcriptional gene regulation, influencing various cellular processes. They act as molecular orchestrators, fine-tuning gene expression to maintain cellular homeostasis. MicroRNAs are short RNA molecules, typically composed of 19–25 nucleotides, and were initially discovered in 1993 [15]. Unlike protein-coding genes, microRNAs do not give rise to proteins themselves; instead, they exert control over the expression of target genes post-transcriptionally. This means that microRNAs regulate the amount of protein produced from a specific gene [16].

**Fig. 1:**
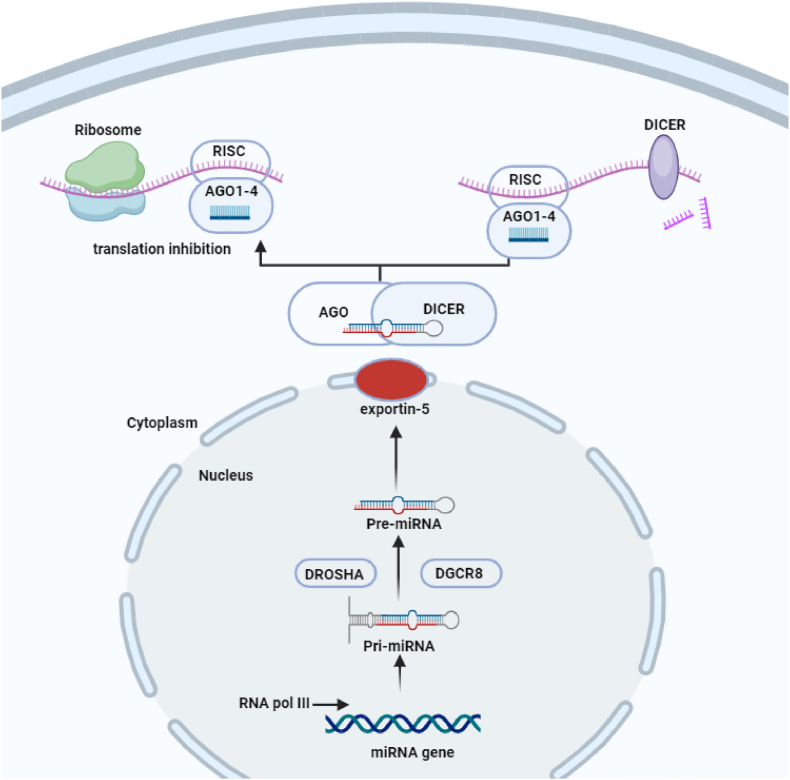
Usual bioformation process of miRNAs. they are transcribed as primary miRNAs (pri-miRNAs) by RNA polymerase II. Microprocessor complex, consists of the RNase III enzyme Drosha and the double-stranded RNA (dsRNA)-binding protein DiGeorge critical region 8 (DGCR8), cleaves the pri-miRNA and releases pre-miRNAs. pre-miRNAs are bound by the export receptor exportin 5 and transported into the cytoplasm. in cytoplasm, the RNase III endonuclease DICER cleaves the pre-miRNA, producing double-stranded miRNAs. MicroRNAs are further processed by Argonaute (Ago) through cleavage to form the miRNA-induced silencing complex (miRISC). This figure was prepared using biorender.

MicroRNAs are transcribed from DNA in the cell nucleus to form primary microRNA (pri-miRNA) transcripts. These pri-miRNAs are processed into precursor microRNAs (pre-miRNAs), which are subsequently exported to the cytoplasm. In the cytoplasm, pre-miRNAs are further processed to mature microRNAs. These mature microRNAs are incorporated into Argonaute proteins to form the RNA-induced silencing complexes (RISCs). MicroRNAs contain a specific sequence called the "seed region,” typically at nucleotide positions 2–8. The seed region is complementary to specific sequences in the messenger RNA (mRNA) molecules of target genes [17]. MicroRNAs recognize and bind to their target mRNAs based on sequence complementarity. Once bound to their target mRNAs, microRNAs guide the RISC complex to the mRNA molecule. The RISC complex can then perform two main regulatory functions: 1. mRNA Degradation: In some cases, the binding of microRNAs to their target mRNAs leads to mRNA degradation, preventing the translation of the mRNA into a functional protein [17]. 2.Translation Inhibition: In other cases, microRNAs inhibit translation by preventing the ribosome, the cellular machinery responsible for protein synthesis, from attaching to the mRNA. By binding to specific target mRNAs, microRNAs fine-tune gene expression levels, ensuring proteins are produced at the right time and in the right amounts [17,18]. This precise regulation is essential for normal cellular functions and development. In Summary, microRNAs serve as critical regulators of gene expression through their involvement in post-transcriptional gene regulation. They achieve this by guiding RNA-induced silencing complexes to their target mRNAs, where they can either trigger mRNA degradation or inhibit translation. This sophisticated molecular orchestra of microRNAs plays a vital role in maintaining cellular homeostasis, and their dysregulation is implicated in various diseases, making them a subject of intense research and therapeutic potential in molecular biology [19] [Fig. 1].

## 3 MicroRNAs with possible diagnostic application

### 3.1 MicroRNA-151a: diagnostic potential and IL-12 receptor β2 regulation in atopic dermatitis (AD)

MicroRNA-151a emerges as a promising diagnostic biomarker for AD, while also playing a crucial role in regulating the IL-12 receptor β2 (IL12RB2), shedding light on potential therapeutic avenues [20]. MicroRNA-151a belongs to miR-28 family [15].

AD is notorious for its clinical heterogeneity and the lack of specific diagnostic markers. MicroRNAs have gained attention as potential diagnostic indicators due to their tissue-specific expression patterns and involvement in disease pathogenesis. MicroRNA-151a, in particular, has exhibited altered expression profiles in AD patients compared to healthy individuals [20]. Its levels are often upregulated in AD, making it a potential diagnostic biomarker. Detection of miR-151a in skin samples or peripheral blood may serve as an adjunctive tool for AD diagnosis, complementing existing clinical criteria. MicroRNA-151a′s involvement in AD extends beyond diagnosis, as it plays a significant role in the downregulation of the IL-12 receptor β2 (IL12RB2) [20]. IL12RB2 is a critical component of the interleukin-12 (IL-12) receptor complex, which is involved in immune responses. Dysregulation of IL12RB2 has been implicated in the pathogenesis of AD. MicroRNA-151a exerts its influence by directly targeting IL12RB2 mRNA, leading to its post-transcriptional downregulation. This interaction results in reduced expression of IL12RB2, potentially altering the responsiveness of immune cells to IL-12 signaling. Given the role of IL-12 in immune regulation, the miR-151a-IL12RB2 axis represents an intriguing avenue for further research into AD immunopathology [15].

MicroRNA-151a′s altered expression pattern in AD patients positions it as a valuable diagnostic tool, aiding in accurately identifying individuals with this chronic skin disorder. Its potential to complement clinical assessments offers a non-invasive and efficient means of diagnosis. MicroRNA-151a′s capacity to regulate IL12RB2 hints at its involvement in immune dysregulation observed in AD. Further exploring this mechanism may provide insights into the intricate immune pathways contributing to AD pathogenesis, opening avenues for targeted therapeutic interventions [20].

In summary, miR-151a emerges as a dual player in AD—offering diagnostic potential and shedding light on immune regulation through IL12RB2 modulation. Its diagnostic utility could enhance early detection and management of AD, while its role in immune pathways presents opportunities for therapeutic innovation. Continued research into miR-151a′s precise mechanisms and clinical applications holds promise for advancing our understanding and treatment of AD.

### 3.2 MicroRNA-155: central role in immune responses in atopic dermatitis (AD)

MicroRNA-155 emerges as a pivotal player in immune responses and Th17 cell differentiation, with promising potential as a diagnostic marker and therapeutic target in AD [21]. It also directly targets negative regulator of T cell activation, Cytotoxic T Lymphocyte Antigen-4 (CTLA-4) [22]. Within the intricate web of immune regulation, miR-155 shines as a multifaceted orchestrator, particularly in the context of AD. It holds a central position in modulating immune responses, making it a compelling subject of study in AD. It is intricately involved in the differentiation and function of immune cells, particularly T cells and dendritic cells. MicroRNA-155 is essential for the generation of Th17 cells, a subset of T helper cells known for their involvement in inflammatory responses [22]. Th17 cells and their signature cytokines, such as interleukin-17 (IL-17), are key players in AD pathogenesis. MicroRNA-155 regulates this intricate immune dance by fine-tuning the expression of genes involved in Th17 cell differentiation and function [21]. MicroRNA-155 promotes Th17 cell differentiation by targeting and modulating the expression of key regulatory genes. This pivotal role in Th17 cell development implicates miR-155 in AD's immune dysregulation. Dysregulated Th17 responses contribute to the chronic inflammation and tissue damage seen in AD-affected skin. By influencing Th17 cell differentiation, miR-155 significantly contributes contributor to AD's immunopathology [23].

MicroRNA-155's importance in AD extends beyond immune regulation, encompassing diagnostic and therapeutic potential. Altered expression of miR-155 has been observed in AD patients [23]. Elevated miR-155 levels in skin samples or peripheral blood can potentially serve as a diagnostic marker, aiding in identifying AD cases. Its specificity to immune responses and its role in AD pathogenesis enhances its diagnostic value, particularly in challenging cases. MicroRNA-155 inhibition presents an exciting avenue for therapeutic intervention. By curbing its activity, researchers aim to mitigate Th17 cell-driven inflammation in AD [24]. Therapies targeting miR-155 may help restore immune balance, reduce chronic inflammation, and alleviate AD symptoms. However, careful consideration of the broader immune effects is necessary to ensure a balanced immune response during treatment [25].

In summary, miR-155 takes center stage in AD's immune orchestra, influencing Th17 cell differentiation and immune responses by targeting the CTLA-4. It's potential as a diagnostic marker promises improved AD diagnosis, while its role as a therapeutic target offers hope for novel treatment strategies. Unraveling the intricacies of miR-155's functions in AD opens doors to a deeper understanding of this complex skin disorder and the development of more precise diagnostic tools and targeted therapies.

## 4 MicroRNAs with possible therapeutic application

### 4.1 MicroRNA-10a-5p: establishing keratinocyte dynamics

MicroRNA-10a-5p, a specific microRNA, emerges as a critical player in the intricate symphony of skin biology, particularly in orchestrating keratinocyte proliferation, differentiation, and skin barrier function. Its dysregulation has significant implications for susceptibility to Atopic Dermatitis (AD). Keratinocytes are the predominant cell type in the epidermis, forming the outermost layer of our skin. MicroRNA-10a-5p has been identified as a regulator of keratinocyte proliferation. It promotes cell division when necessary, aiding in the repair of skin damage and the replacement of old cells. This regulatory function ensures the continuous renewal of the epidermal layer. Equally crucial is miR-10a-5p′s role in keratinocyte differentiation [26]. As keratinocytes migrate upwards through the epidermal layers, they transform to become fully functional, protective skin cells. MicroRNA-10a-5p helps guide this differentiation process, ensuring the epidermis maintains its integrity and barrier function. The skin barrier is the body's first line of defense against external threats, such as allergens, microbes, and environmental irritants. MicroRNA-10a-5p plays a pivotal role in maintaining this barrier. Dysregulation of miR-10a-5p can disrupt the delicate balance of keratinocyte dynamics, impairing skin barrier function [26]. MicroRNA-10a-5p target and inactivates the mRNA of HAS3. HAS3 is a positive regulator of proliferation and migration of keratinocytes related to damages [26]. A compromised barrier allows allergens and irritants to penetrate the skin more easily, triggering immune responses characteristic of AD. Research suggests that individuals with AD often exhibit altered miR-10a-5p expression patterns. This altered miRNA expression contributes to the breakdown of the skin barrier and the onset of AD symptoms, such as pruritus, erythema, and eczematous lesions. MicroRNA-10a-5p′s role in regulating genes involved in barrier formation and immune responses positions it as a crucial molecular determinant in AD susceptibility [15].

In summary, miR-10a-5p is a molecular conductor in the intricate orchestra of skin biology, directing the harmonious interplay of keratinocyte proliferation, differentiation, and skin barrier function. Dysregulation of this microRNA can lead to a discordant note in this symphony, compromising the skin barrier's integrity and increasing susceptibility to Atopic Dermatitis. Understanding and targeting miR-10a-5p may hold promise for innovative therapeutic interventions in AD and related skin disorders.

### 4.2 MicroRNA-29b: regulating keratinocyte apoptosis and barrier integrity

MicroRNA-29b, a microRNA of particular interest in the context of skin biology, plays a pivotal role in regulating keratinocyte apoptosis and maintaining epithelial barrier integrity. Its influence extends to the severity of Atopic Dermatitis (AD) [27]. It directly targets BCL-2-Like Protein 2 and mediates IFN-γ related apoptosis in keratinocytes [28]. Keratinocyte apoptosis is a finely tuned process that ensures the removal of damaged or surplus skin cells. MicroRNA-29b is a key regulator in this intricate mechanism. This microRNA acts as an anti-apoptotic factor, and helps to prevent excessive keratinocyte cell death. By targeting specific genes involved in apoptosis, this microRNA ensures that the epidermis maintains an optimal balance between cell death and cell proliferation. This balance is essential for skin homeostasis and repair [27].

MicroRNA-29b′s role in keratinocyte biology has a direct impact on epithelial barrier integrity [27]. Dysregulation of miR-29b can disrupt the normal life cycle of keratinocytes, leading to aberrant barrier function. This disruption allows for increased permeability, facilitating the entry of allergens and irritants [29]. The compromised barrier contributes to the characteristic features of AD, including pruritus, erythema, and eczematous lesions. Numerous studies have shown an association between altered miR-29b expression and the severity of AD. Reduced miR-29b levels are often observed in individuals with more severe AD symptoms. This suggests that miR-29b may serve as a biomarker for AD severity and a potential target for therapeutic interventions to restore barrier function [30].

In summary, miR-29b′s role in skin biology is multifaceted. It acts as a guardian against excessive keratinocyte apoptosis, ensuring the proper balance of cell turnover. Additionally, it plays a crucial role in maintaining epithelial barrier integrity, a critical defense mechanism for the body. Dysregulation of miR-29b contributes to the development and severity of Atopic Dermatitis, making it a promising focus for future research and therapeutic strategies aimed at alleviating AD symptoms and restoring skin barrier function.

### 4.3 MicroRNA-124: anti-inflammatory effects in atopic dermatitis (AD)

MicroRNA-124, a remarkable microRNA, emerges as a key player in AD due to its potent anti-inflammatory properties. It exerts its effects by targeting and inhibition of p65 subunit of NF-κB signaling pathway and holds significant promise as a therapeutic target for AD [31]. MicroRNA-124 stands out in the context of AD for its ability to modulate the NF-κB signaling pathway, a central regulator of inflammation. One of the primary mechanisms through which miR-124 exerts its anti-inflammatory effects is by directly targeting and inhibiting components of the NF-κB pathway [31]. NF-κB is a transcription factor that is pivotal in initiating the inflammatory response. By downregulating NF-κB activity, miR-124 effectively dampens the expression of pro-inflammatory cytokines, chemokines, and adhesion molecules. This, in turn, reduces the recruitment and activation of immune cells involved in the inflammatory cascade, mitigating the inflammatory response seen in AD [31]. It should be noted that the miR-124 could be inhibited by TNF-α and IFN-γ.

MicroRNA-124's unique ability to quell inflammation makes it a promising candidate for AD therapeutics. The chronic inflammatory nature of AD is a primary driver of its pathogenesis and clinical manifestations [32]. MicroRNA-124's ability to counteract inflammation positions it as a potential therapeutic target. By harnessing the anti-inflammatory power of miR-124, researchers aim to develop innovative treatment strategies that address the root cause of AD, relieving symptoms such as pruritus, erythema, and eczematous lesions. Beyond its anti-inflammatory role, miR-124 has been associated with tissue repair and regeneration [31]. In AD, where skin barrier disruption is a hallmark feature, promoting skin healing is of utmost importance. MicroRNA-124's dual capacity to reduce inflammation and enhance tissue repair makes it an even more appealing target for therapeutic interventions [15].

In summary, miR-124 emerges as a critical regulator in the pathogenesis of AD, primarily through its anti-inflammatory actions via the NF-κB signaling pathway. As the development of targeted therapies for AD advances, miR-124 holds significant promise as a therapeutic target. Harnessing its anti-inflammatory potential may pave the way for more effective treatments that not only alleviate AD symptoms but also address the underlying mechanisms driving this complex skin disorder.

### 4.4 MicroRNANA-143: countering allergic inflammation in atopic dermatitis (AD)

MicroRNA-143 emerges as a pivotal regulator in the context of AD, primarily due to its ability to target the IL-13 receptor alpha 1 (IL-13Ra1) and its potential to mitigate the symptoms of this allergic skin disorder [33]. MicroRNA-143 plays a crucial role in countering allergic inflammation in AD through its interaction with the IL-13Ra1. IL-13 is a key player in the immune responses associated with AD. It promotes allergic inflammation and contributes to the disruption of the skin barrier. MicroRNA-143's noteworthy function is its ability to target and downregulate the expression of IL-13Ra1 [33]. By doing so, it acts as a natural brake on the IL-13 signaling pathway. This dampening of IL-13 signaling is significant, as it curtails the production of pro-inflammatory cytokines and chemokines, ultimately reducing the inflammatory response within the skin [34].

MicroRNA-143 offers a promising avenue for alleviating the symptoms of AD and potentially modifying the disease course. In individuals with AD, there is often a dysregulation in the expression of various miRNAs, including a decrease in miR-143 levels [33]. This deficiency contributes to heightened inflammation and disease severity. Researchers are exploring strategies to restore miR-143 levels in AD patients. By doing so, it is anticipated that the excessive IL-13 signaling, which drives the allergic inflammation seen in AD, could be controlled. This restoration could potentially lead to ameliorating AD symptoms such as pruritus, erythema, and eczematous lesions. MicroRNA-143's role in targeting IL-13 signaling pathways holds significant therapeutic implications. Novel therapies aimed at replenishing miR-143 or modulating its activity may offer a more precise and targeted approach to managing AD. Such interventions could reduce the reliance on broad immunosuppressive treatments, minimizing side effects and enhancing the overall quality of life for individuals with AD [35].

In summary, miR-143 plays a pivotal role in AD by countering allergic inflammation by targeting of the IL-13Ra1. Restoring miR-143 levels hold promise as a therapeutic strategy to mitigate AD symptoms and address the underlying mechanisms driving this chronic skin disorder. The research surrounding miR-143 underscores its potential to transform the landscape of AD treatment, offering hope for improved outcomes and enhanced patient well-being.

### 4.5 MicroRNA-146a-5p: immune regulation in atopic dermatitis (AD)

MicroRNA-146a-5p, a master regulator of immune responses, emerges as a key player in the pathogenesis of AD. Its multifaceted role includes immune regulation, particularly in the NF-κB pathway, and a noteworthy correlation with IgE levels in AD. MicroRNA-146a-5p is a pivotal microRNA with a pronounced role in immune regulation within the context of AD, primarily by modulating the NF-κB pathway [36]. The NF-κB pathway is a central regulator of inflammation and immune responses. In AD, this pathway is often hyperactivated, leading to an exaggerated immune response and the characteristic inflammatory symptoms. MicroRNA-146a-5p functions as a negative regulator of NF-κB signaling [37]. It achieves this by targeting key components of the pathway, including interleukin-1 receptor-associated kinase 1 (IRAK1) and tumor necrosis factor receptor-associated factor 6 (TRAF6) and CARD-10. Human CCL5 is also identified as a target of miR-146a-5p [36]. By downregulating these components, miR-146a-5p exerts a dampening effect on NF-κB activation, reducing the production of pro-inflammatory cytokines and chemokines. This regulatory role is crucial in mitigating the inflammatory cascade observed in AD [37].

MicroRNA-146a-5p levels also correlate with IgE levels, a hallmark feature of AD. Elevated IgE levels is a common feature of AD and contribute to the allergic inflammation seen in this condition. Studies have revealed that miR-146a-5p expression inversely correlates with IgE levels in AD patients [38]. In essence, higher miR-146a-5p levels are associated with lower IgE levels. This correlation suggests that miR-146a-5p may play a role in modulating IgE production or signaling pathways involved in IgE regulation. Understanding and harnessing this correlation may offer novel therapeutic strategies to reduce IgE-mediated allergic responses in AD [39].

In summary, miR-146a-5p is a central player in the immune dysregulation observed in AD. Its ability to suppress NF-κB activation and its correlation with IgE levels highlight its significance in the pathogenesis of AD. Harnessing the regulatory potential of miR-146a-5p holds promise for developing targeted therapies aimed at controlling inflammation and allergic responses in AD. Further research into the precise mechanisms by which miR-146a-5p influences IgE levels may unveil innovative approaches to managing this chronic skin disorder and improving the quality of life for affected individuals.

### 4.6 MicroRNA-223: prenatal exposure and immune modulation in atopic dermatitis (AD)

MicroRNA-223emerges as a fascinating player in AD, particularly in the context of prenatal exposure to tobacco smoke and its potential influence on regulatory T cells (T-reg cells) and AD risk [40]. Understanding the environmental factors contributing to AD risk is vital, and prenatal exposure to tobacco smoke has surfaced as an intriguing avenue of investigation. Emerging research suggests that prenatal exposure to tobacco smoke may increase the risk of AD development in offspring. This risk, however, is not uniform and varies among individuals. MicroRNA-223has recently entered the spotlight as a potential molecular mediator of this phenomenon [41]. MicroRNA-223is a microRNA with diverse roles in immune regulation, and its implications in AD are worth noting. MicroRNA-223plays a pivotal role in modulating immune responses. It influences the differentiation and function of regulatory T cells (T-reg cells), a subset of immune cells tasked with maintaining immune balance and preventing excessive inflammation. T-reg cells are critical in curbing the immune responses that drive AD pathology [42]. The connection between miR-223, prenatal smoke exposure, and AD risk becomes more apparent when examining miR-223impact on T-reg cells.

MicroRNA-223appears to modulate the suppressive function of T-reg cells. When miR-223levels are elevated due to prenatal smoke exposure or other factors, it may disrupt T-reg cell function. This disruption can tip the balance towards heightened immune activation and inflammation, potentially contributing to AD susceptibility [43]. The interplay between miR-223, prenatal exposure to tobacco smoke, and T-reg cells carries significant implications. Prenatal smoke exposure-induced changes in miR-223levels may contribute to an imbalance in immune regulation, favoring pro-inflammatory responses. This imbalance aligns with the immune dysregulation observed in AD. As a result, individuals exposed to prenatal tobacco smoke and carrying variations in miR-223regulation may face an increased risk of developing AD [44].

**Fig. 2:**
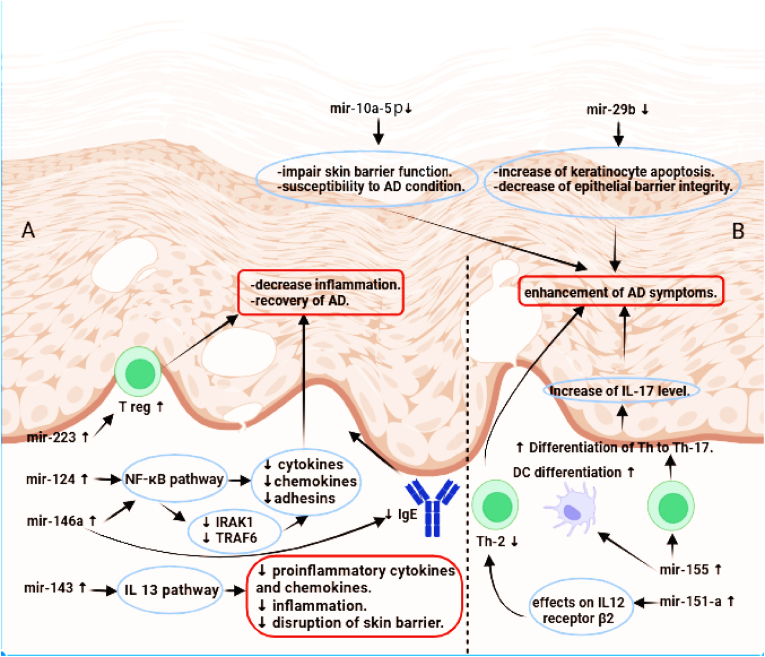
Mechanisms of action of microRNAs in Atopic Dermatitis. This figure was prepared using biorender.

In summary, miR-223's role in AD extends beyond immune modulation to encompass the intriguing link between prenatal tobacco smoke exposure, immune balance, and AD risk. Research into miR-223sheds light on the complex interplay of genetic, environmental, and immune factors contributing to AD development. Understanding these intricate connections provides valuable insights into potential strategies for AD prevention and personalized treatments that target miR-223-related pathways [Fig. 2].

## 5 Conclusion

In recent years, the study of microRNAs (miRNAs) has unveiled a new dimension in our understanding of Atopic Dermatitis (AD). These tiny molecules have emerged as molecular orchestrators in the intricate landscape of AD pathogenesis. Through the post-transcriptional regulation of gene expression and modulation of immune responses, microRNAs exert profound effects on the development and progression of AD. they affect the activities of Th-17 and T-reg cells, cytokine signaling pathways, NF-κB-related inflammation, and finally, aberrant skin barrier function. Several microRNAs, including miR-10a-5p, miR-29b, miR-124, miR-143, miR-146a-5p, miR-151a, miR-155, and miR-223, have been scrutinized for their roles in AD [15,41]. Each microRNA brings a unique set of functions, collectively influencing keratinocyte dynamics, immune regulation, inflammatory responses, and even prenatal exposure effects. Their intricate involvement in AD reflects the multifaceted nature of this skin disorder. MicroRNA-10a-5p emerges as a key player in keratinocyte proliferation and differentiation, whereas miR-29b delicately maintains keratinocyte apoptosis and barrier integrity. MicroRNA-124 stands out for its anti-inflammatory prowess, targeting the NF-κB signaling pathway, and presenting a promising therapeutic target. MicroRNA-143 counteracts allergic inflammation by targeting the IL-13 receptor alpha 1, offering a potential route to alleviate AD symptoms. In the realm of immune regulation, miR-146a-5p takes center stage, orchestrating the NF-κB pathway and correlating with IgE levels in AD. MicroRNA-151a′s diagnostic potential shines through its role in regulating IL-12 receptor β2 (IL12RB2), providing insight into AD diagnosis and management. MicroRNA-155 occupies a central role in immune responses and Th17 cell differentiation, making it a diagnostic marker and a therapeutic target of interest. Lastly, miR-223's connection to prenatal smoke exposure and T-reg cells adds a layer of complexity to understanding AD susceptibility [23].

In conclusion, microRNAs are emerging as fundamental players in the intricate conformity of AD pathogenesis. The knowledge gained from these small but potent molecules opens up new horizons for understanding, diagnosing, and treating AD. Consequently, mechanism-driven therapy for this refractory dermatological illness, for which there are currently very few therapeutic choices, would surely be made easier with the experimental confirmation of AD-associated miRNAs and their downstream mediators as druggable targets. While the same miRNA may act differently in different tissues, research should also be done to optimize the delivery of miRNA mimics or inhibitors in a cell-type-specific manner. Finally, Due to the small amount of proven information available about the therapeutic effects and diagnostic usage of microRNAs, we need more research on the exact effects and mechanisms of action of these molecules in various levels of Atopic Dermatitis.

## Ethics approval and consent to participate

Not applicable.

## Consent for publication

Not applicable.

## Availability of data and materials

Not applicable. All data are available as addressed references.

## Funding

Not used.

## CRediT authorship contribution statement

**Mahsa Khosrojerdi:** Writing – review & editing, Writing – original draft, Software, Methodology, Investigation. **Farahzad Jabbari Azad:** Writing – original draft, Supervision, Conceptualization. **Yaser Yadegari:** Visualization, Validation, Data curation. **Hamid Ahanchian:** Supervision, Data curation, Conceptualization. **Amir Azimian:** Writing – review & editing, Writing – original draft, Visualization, Validation, Supervision, Software, Resources, Project administration, Methodology, Investigation, Formal analysis, Data curation, Conceptualization.

## Declaration of competing interest

The authors declare that they have no known competing financial interests or personal relationships that could have appeared to influence the work reported in this paper.
